# Retrospective Assessment of Human–Chemical Interactions in Health-Disparity Populations: A Process Evaluation of Life History Calendars

**DOI:** 10.3390/ijerph191912397

**Published:** 2022-09-29

**Authors:** Michael Anastario, Olivia Ceavers, Paula Firemoon, Nezahualcoyotl Xiuhtecutli, Ana Maria Rodriguez

**Affiliations:** 1 Robert Stempel College of Public Health & Social Work, Florida International University, Miami, FL 33174, USA; 2 Fort Peck Community College, Poplar, MT 59255, USA; 3 Farmworker Association of Florida, Apopka, FL 32703, USA

**Keywords:** life-history calendar, people who use injection drugs, agricultural workers, ethics, testimonies

## Abstract

Life-history calendars (LHCs) can produce retrospective data regarding numerous events, exposures, and sequences that have occurred across participants’ lifespans. In this mixed-quantitative-and-qualitative-methods study, processes of LHC administration were evaluated in two populations experiencing health disparities: foreign-born agricultural workers (n = 41) and Indigenous people who used injection drugs (IPWIDS) (n = 40). LHC administrator and participant perspectives were elicited during follow-up survey activities. In both agricultural workers and IPWIDs, over half of participants reported that the LHC made it easier to remember things about the past, and participant age was associated with cumulative experience in different domains of interest. Qualitative findings suggested that data-collector training and the development of concise interview guides are critical for improving LHC data quality. Participants described ethical themes, including utilitarian, cathartic, and reflective aspects, of LHC participation. Future iterations of the LHC may benefit from providing free-form and open-ended spaces for participants to reflect on the LHC activity following LHC administration.

## 1. Introduction

The life-history calendar (LHC) is a visual, grid-format timeline for collecting data on multiple domains of interest over a defined time-period within a research participant’s lifespan [1,2]. LHCs are used to facilitate retrospective data collection regarding the timing, occurrence, and sequencing of life events based on participants’ self-reports [3]. The method is thought to enhance retrospective reporting by reflecting the structure of autobiographical reporting [4]. While LHCs have been employed by researchers to collect retrospective data regarding numerous events, exposures, and sequences that have occurred across participants’ lifespans, little attention has been paid to the process of LHC administration. In this paper, we evaluate processes of LHC administration concerning different health issues occurring in two populations experiencing health disparities.

LHCs are useful tools for collecting data on sequential phenomena over defined periods of time. While they have been used in the social sciences for this purpose (e.g., to understand sequences of significant life events), LHCs are amenable to capturing phenomena relevant to public health. For example, in the environmental health sciences, the concept of the exposome has been posited as “the cumulative measure of environmental influences and associated biological responses throughout the lifespan including exposures from the environment, diet, behavior, and endogenous processes” [5,6]. Developing social-scientific methods to elicit information regarding human–chemical interactions over the lifespan may be one way in which everyday knowledge of exposures can be articulated with more classical methods of exposure assessment. The use of LHCs may simultaneously help to facilitate an understanding of life courses in populations experiencing health disparities, particularly in research that examines how lived experiences and trajectories influence health over the life course [7]. When health-disparity populations experience long-term exposures to harmful substances, narratives containing knowledge of these exposures can be difficult to translate in ways that make these narratives accessible to Western science. Agricultural workers and people who use injection drugs both experience health disparities and exposures to harmful substances. LHCs have been used to study occupational exposures in agricultural workers and histories of substance use among people with chemical dependencies, but limited attention has been paid to LHC administration. We briefly describe the use of LHCs in both populations separately.

LHCs have been particularly useful in measuring agrichemical exposures relative to periods of the life course where it would otherwise be difficult to conduct an assessment. For example, Quandt et al. (2020) used information collected via LHC as a proxy to determine before-and-after birth exposure to pesticides of Latinx children in farmworker families [8]. In Nicaragua, Rodriguez et al. (2012) used icon-calendar-based forms to interview parents about children’s pre- and post-natal pesticide exposures to develop cumulative indices of exposure, identifying a total of 47 pesticides reported and concluding that a retrospective construction of cumulative pesticide-use indices could improve quantitative exposure assessment in developing countries [9]. A similar study in Costa Rica used an icon-calendar form to assess pesticide exposure during the perinatal period and found an elevated risk of childhood leukemia in association with parents’ occupational exposures during the perinatal period and the first year of the child’s life [10]. There is a need for more methodological research elucidating how LHCs are applied in studies that collect background social information, such as occupational histories [2]. Elucidating the process of LHC administration may reveal additional elements of the methodology that should be considered and planned for in studies where LHCs are used. 

In the context of substance-abuse research, the LHC is distinct from follow-back timelines (typically time-delimited) or interpretive life-history approaches to substance use in that it can be used to quantify lifetime-use sequences in communities, potentially elucidating community-level trends in substance use over time. The LHC has been used in a variety of substance-use-related studies that include alcohol, marijuana, opioids, and other injection drugs [11,12,13,14,15,16,17,18,19,20,21,22,23,24,25]. In a study of adult opioid users, the LHC was found to provide rich data regarding life events, living situation, personal experience, and developmental periods relevant to the individual’s substance-use experience [23]. Distinct from follow-back timelines, LHCs may aid in the measurement of substance use across the participants’ entire lifespans. 

While LHCs have been employed to retrospectively assess sequential phenomena among agricultural workers and people who use substances, limited attention has been paid to LHC administration, the experiences of participants and administrators of LHCs, and ethical issues concerning LHCs. Evaluating the process of LHC administration will inform the development and implementation of LHCs used in studies with populations experiencing health disparities. In this study, we report on similar process-evaluation measures that were collected across two separate pilot studies conducted with agricultural workers and people who used injection drugs. 

## 2. Materials and Methods

### 2.1. Setting and Design

This is a mixed-quantitative-and-qualitative-methods study of the process of the administration of LHCs in two populations experiencing health disparities that narrate exposure to harmful substances across the lifespan. The data are derived from two separate pilot studies with similar approaches to evaluating the process of administering LHCs. The first pilot study was conducted with 41 foreign-born agricultural workers living in South Florida, and the second pilot study was conducted with 40 Indigenous people who used injection drugs. Both pilot studies were conducted using a community-engaged research framework, which included working with members of community agencies to describe the research, recruit participants, and disseminate results back to the community. Both pilot studies contained follow-up surveys that elicited participant perspectives regarding LHC activity. While findings that employ social-sequence analysis to analyze human–chemical interactions derived from LHC data are described/being published elsewhere [26], this current study is concerned with evaluating the process of LHC administration by leveraging data collected across both pilot studies. Each pilot study is described briefly below.

The pilot study with agricultural workers took place over a 3-month period (March–May 2021) in Immokalee, Florida. Researchers at Florida International University (FIU) used a community-engaged research process [27,28] to set research priorities, design the study protocol, collect data, and disseminate results in collaboration with the Farmworker Association of Florida (FWAF), a statewide, grassroots, community-based, non-profit farmworker-membership organization with five offices located throughout rural Florida. One of the FWAF’s offices is in Immokalee, a predominantly Hispanic (70.8%) community located in Collier County, Florida [29]. Prior to beginning the study, researchers and community organizers attended a monthly FWAF meeting in Immokalee to introduce the research team, describe the overall aims, explain the eligibility criteria, and answer any questions or concerns of the potential local participants. All presentations and data collection occurred in the Spanish language. FWAF employees recruited participants and introduced them to data collectors. Eligible participants were agricultural workers of Mesoamerican descent with lifetime work experience in agriculture. In total, 42 people were recruited, but one participant was excluded from the analyses when the LHC activity revealed that the participant was born in the United States. Interviews lasted 38–105 min and took place in a private room in a church (which shared office space with the FWAF Immokalee office). Interview incentives included USD 40 in cash. Ethical approval for the study was given by the Florida International University Institutional Review Board (IRB). Results from the initial pilot study were presented back to community members at a monthly meeting.

The pilot study with Indigenous people who used injection drugs (IPWIDs) took place over a 6-month period (October 2021–April 2022) on the Fort Peck Indian Reservation in northeastern Montana. Researchers at FIU, Montana State University, and Fort Peck Community College collaborated to conduct the study as part of a larger, ongoing community-based participatory research project with the Fort Peck Tribes. Eligibility criteria included currently injecting drugs or previously injecting substances for ≥12 months, being a registered member of a federally recognized tribe or an associate tribal member, and being older than 13 years of age. The study’s only exclusion criterion was being currently incarcerated and/or in police custody at the time of the study. The study’s tribal director recruited participants, and in some instances participants were smudged with sage upon entering and leaving the data-collection facility. In total, 41 participants were recruited, but one participant was excluded for not being a tribal member. All study participants received a USD 50 gift card for participating in the interview. Human Subjects approval was obtained from the Fort Peck IRB and the Florida International University IRB.

#### Life-History Calendars

Both pilot studies used LHCs to collect data on human–chemical interactions over the lifespans of participants in single sittings. In both populations, exposure to harmful substances over the lifespan was a common experience. In agricultural workers, the LHC was focused on collecting occupational histories and sequences of agrichemicals used. In IPWIDs, the LHC was focused on injection practices and sequences of substances injected. While the focus and scope of both studies varied, there were similar questions asked regarding the process of LHC administration. In both studies, participants were asked nine identical questions regarding the process of LHC administration (further described below in “Assessments by LHC administrators” and “Assessments by LHC participants”). In both studies, a semi-structured interview guide facilitated the completion of a baseline survey, the LHC, and a follow-up survey. The LHC for agricultural workers was administered using paper only, with 1 participant completing an electronic LHC to test-run the modality of administration. The later study conducted in IPWIDs randomized participants to either an electronic (n = 20) or paper-based (n = 20) modality of administration. Paper-based LHCs in both studies appeared on two 30 “×42” pieces of paper. Colored pencils were used to delineate phases of life described by the participant, and pens were used to annotate responses on the LHC forms. The follow-up survey contained questions for both the interviewer and participant regarding the LHC activity that just took place. 

### 2.2. Measures

Demographic characteristics. In both agricultural workers and IPWIDs, the demographic characteristics of participants were collected during the baseline survey. In agricultural workers, information was collected on self-reported gender identity, whether the participant spoke an Indigenous language in addition to Spanish, the age of the participant, and country of origin prior to migrating to the United States. In IPWIDs, information was collected on self-reported gender identity, the age of the participant, and tribal registration status.

Assessments by LHC administrators. Immediately following completion of the LHC, administrators were asked five brief questions to evaluate their experience administering the LHC. This included documenting the number of minutes it took for a given participant to complete the LHC and to self-assess the ease of LHC administration with the participant using a 10-point global assessment ranging from 1 (asiest) to 10 (most difficult). Three open-ended items were included for LHC administrators to provide commentary regarding problems they experienced, perceptions of actions that would have made administering the LHC easier for a given participant, and whether there were any aspects of the LHC that were useful for the participant. These final items were included to allow for direct suggestions of LHC administrators to be incorporated into future drafts of LHCs that would be conducted with a similar population.

Assessments by LHC participants. Following completion of the LHCs, participants were asked four brief questions regarding their experience with the LHC. Participants were asked how they felt after completing the diagram and what they thought about the LHC. Open-ended responses were recorded by the administrator. Participants were then asked, if they had to choose between this information being recorded on a computer or a piece of paper, which would they prefer? Participants were also asked to evaluate ease of recall using a three-point response set, indicating whether seeing parts of their past represented on the LHC made it easier (1), had no effect (2), or made it harder (3) to remember events about their life. 

Occupational experiences and agrichemical exposures in agricultural-worker LHCs. While the LHC included multiple domains of measurement, the primary domain of interest for this study concerned the measurement of agrichemical exposures relative to occupation, a section of the interview which took up the entire second page of the LHC. Participants delineated their occupational experiences over the life course and were subsequently asked to respond to an inventory of questions documenting potential agrichemical exposures relative to each occupation. Responses were plotted using the color code of the occupational activity. For each occupation that required working on manmade terrestrial ecosystems, participants were asked to recall years of involvement in: pesticide mixing, insecticide application, herbicide application, fungicide application, fertilizer application, working in a field where someone else was applying pesticide, and application of an unknown chemical. Follow-up questions for a positive response included the name(s) of the agrichemical(s) used and whether personal protective equipment (PPE) was used. The cumulative number of person-years worked (range: 9–62) and the cumulative number of person-chemical-exposure-years reported (range: 5–185) were compared relative to LHC-participant and -administrator characteristics.

Polysubstance use in IPWID LHCs. LHC forms were used to measure substance use over the lifespan. Individuals were first asked to explain where they had lived since birth, followed by the year the participant began injecting substances and the individual parts of the body (arms, legs, neck, groin, fingers/toes, other) that the participant had injected substances into during a given year of life. After establishing this information to serve as a potential anchor, an inventory of substances used was plotted, by year, over the lifespan. The items in the substance-use inventory were based on substances previously described in formative qualitative research with IPWIDs [30]. Participants were asked to indicate the years of life they used: alcohol, methadone, Suboxone^®^, buprenorphine, heroin, other opiates/analgesics, barbiturates, sedatives/hypnotics/tranquilizers, cocaine, amphetamines, methamphetamines, cannabis, hallucinogens, inhalants, speedballs (heroin and cocaine mixed together), goofballs (heroin and speed mixed together), injected energy drinks, injected tap water, injected saline solution, and other substances. Other substances reported included varying definitions of speedballs (typically variations of opioids and methamphetamines). The total number of substances used over the lifetime (range: 3–11) and the cumulative person-polysubstance-years of use (range: 19–147) were calculated for each participant. To compare results between modalities of administration, ever having used a substance (0 = never, 1 = at least once) and the number of years of reported use were recorded and calculated for each individual substance derived from the LHC.

### 2.3. Data Analysis

The quantitative analyses included examination of central tendency and dispersion for the follow-up survey items. Analysis of variance was used to evaluate the duration of the LHC and ease-of-administration outcomes in relation to potential demographic characteristics of LHC participants, as well as documentation of occupational-experience items in relation to LHC-participant and -administrator characteristics. Pearson’s chi-squared test was used to evaluate whether participant self-assessment of the LHC making it easier to remember varied with the demographic characteristics of LHC participants. For the LHC modality tests, substance-use variables were compared between participants completing the LHC using the electronic mode (n = 20) versus the paper-based mode (n = 20). Fisher’s exact test was used to evaluate differences in the detection of ever having used a single substance, and the Wilcoxon rank-sum test was used to evaluate differences in the years of reported use for a given substance between modes of LHC administration. 

Qualitative analysis was conducted separately for two sets of interview data: comments provided by LHC administrators in the follow-up survey and open-ended responses from participants in the follow-up survey. Within each set, text-response segments were coded using an inductive analytic procedure to reduce the text segments into axial codes [31]. Then, axial codes were used by a separate coder to recode the original text segments. For the agricultural worker qualitative codes, overall inter-coder agreement was 82% and Cohen’s kappa was 0.76 across the pooled text segments. For the IPWID qualitative codes, overall inter-coder agreement was 100%, and Cohen’s kappa was 1.0 across the pooled text segments. These values fell within a substantial range [32,33]. Axial codes were used to structure the presentation of qualitative results. 

## 3. Results

The final analytic sample of agricultural workers included 27 women and 14 men with an average age of 43 years. There were 25 monolingual Spanish speakers and 16 individuals who spoke an Indigenous language (Mam, Nahuatl, Zapotec, Mixteco, Otomi, and Chinanteco) in addition to Spanish. The final analytic sample of IPWIDs included 22 men and 18 women with an average age of 38 years. Thirty-five participants were registered members of a federally recognized tribe, and five were associate tribal members.

### 3.1. Minutes to Complete and Ease of Administration

In agricultural workers, LHCs took on average 66 min (standard deviation (SD) = 19.5) to complete. LHCs took 11.7 min longer to complete for individuals who spoke an Indigenous language in addition to Spanish (F = 3.7, df = 1, *p* = 0.06) (Table 1). LHC administrators assessed overall ease of administration at 4.8 (SD = 2.8) on the scale ranging from 1 = easiest to 10 = most difficult. LHC administrators assessed individuals from Guatemala as more difficult to interview in comparison to individuals from Mexico (F = 2.7, df = 2, *p* = 0.06) (Table 1). Following LHC completion, 22 participants (54%) reported that the LHC made it easier to remember things about their past, 16 (39%) reported that it had no effect, and 3 (7%) reported that it made things more difficult to remember. 

In IPWIDs, LHCs took on average 46.5 min (SD = 15.1) to complete. LHC administrators assessed overall ease of administration at 3.0 (SD = 2.2). There were no appreciable differences in duration of administration or LHC administrator-assessed ease of administration relative to the demographic characteristics of participants. Following LHC completion, 26 participants (65.0%) reported that the LHC made it easier to remember things about their past, 9 (22.5%) reported that it had no effect, and 5 (12.5%) reported that it made things more difficult to remember (Table 1).

### 3.2. Cumulative Person-Years of Human–Chemical Interactions Documented

In agricultural workers, more cumulative person-years of labor were documented for participants who spoke an Indigenous language in addition to Spanish (F = 6.7, df = 1, *p* = 0.013), individuals ≥60 years of age (F = 27.3, df = 1, *p* < 0.001), and individuals from Mexico (F = 3.6, df = 2, *p* = 0.035) (Table 2). More cumulative person-years of exposure to agrichemicals were reported in individuals ≥60 years of age (F = 4.5, df = 1, *p* = 0.041) and in individuals from Honduras and Guatemala (F = 10.7, df = 2, *p* < 0.001). 

In IPWIDs, more cumulative person-polysubstance-years of use were documented in individuals ≥40 years age (F = 15.3, df = 1, *p* > 0.001) (Table 2). The total number of substances ever used did not vary relative to participants’ demographic characteristics.

### 3.3. Modality of Administration

In agricultural workers, 34 participants (83%) reported that they would prefer to complete the LHC by paper and 7 (17%) reported preferring to complete it by computer. In IPWIDs, 18 participants (45.0%) reported that they would prefer to complete the LHC by paper, 18 (45.0%) reported preferring to complete it by computer, and 4 (10.0%) reported either computer or paper.

In the modality test that was conducted among IPWIDs, administrators evaluated the electronic modality of administration as easier to administer (mean = 2.4, SD = 1.6) in comparison to the paper-based mode (mean = 3.6, SD = 2.6, Z = 1.7, *p* = 0.093). No significant differences were observed in substance use by modality of LHC administration (Table 3), but more years of Suboxone^®^ use was reported on the paper-based modality (mean = 1.0, SD = 1.6) in comparison to the electronic modality of LHC administration (mean = 0.3, SD = 0.5, |Z| = 1.8, *p* = 0.069) (Table 3).

### 3.4. Qualitative Findings—Agricultural Workers

Themes that emerged from the LHC administrator assessments included comments regarding the LHC instrument, perceptions of participants being resistant to questions and/or withholding information, and perceptions of how participants’ individual characteristics affected LHC administration. Some LHC administrators suggested that personal familiarity with agrichemical names mentioned by participants would have been helpful prior to administering the interview, particularly when participants had trouble remembering the exact names of agrichemicals they administered at earlier points in their life prior to migrating (e.g., referring to Gramoxone (paraquat) as “gamasan” during the interview). Administrators emphasized the need for more concise interviewer instructions for all modules, and some struggled with open-ended sections of the interview, particularly the sections where participants free-listed information. It was cumbersome for administrators to document information between two separate LHC forms during the interview. While the one electronic LHC that was administered had the unique effect of the participant repeatedly correcting the administrator’s spelling of places/names, the electronic LHC administrator produced numerous errors in documenting comments, and trouble was experienced in scrolling through domains (vertically) and years (horizontally) on the computer screen. One strength of the paper-based LHC was its ability to provide color-based visual cues for participants. Sometimes, as participants remembered additional details later in the interview, administrators had to go back to earlier sections of the LHC and modify data based on participants recalling lifetime events as the interview proceeded. Finally, administrators described the colored pencils as useful for delineating phases of life horizontally on the LHC but emphasized the need to write explanations or additional details in pen (instead of with colored pencils). 

Administrators often perceived differences in the process of LHC administration relative to the demographic characteristics of participants. For older participants with long histories of lifetime work experiences, administrators sometimes found it difficult to obtain clear responses in the timing of labor and agrichemical exposures, whereas administrators described greater ease collecting specific dates for these events from younger workers. Individuals whose primary language was an Indigenous language (typically Mam speakers from Guatemala) were sometimes described as having trouble recounting their early-life/pre-migration agrichemical exposures. During the interview, several participants disclosed that they were illiterate, which some administrators perceived as making the LHC less interactive and more difficult to administer. Finally, administrators perceived resistance to questions regarding occupational injuries and agrichemical exposures that occurred in the US. Administrators described this as being linked to fear of potential retaliation by an employer and/or the projected machismo/masculinity of male agricultural workers. On some occasions, the administrator had to re-administer the agrichemical recall items after the participant admitted to withholding information during the interview.

Themes that emerged from the LHC-participant assessments included: emotions, perceptions, and reactions to the process of administering the LHC; learning something about their own exposure to agrichemicals through the reflective act of completing the LHC; and feeling that their experiences were valued and contributed to the greater good because of participating in the study. Participants generally described the LHC activity as being easier than they imagined, although some expressed difficulty with remembering the names of agrichemicals used. No participant expressed discomfort or that the activity brought up painful memories. Conversely, several expressed a sense of catharsis. One participant remarked: “I feel that I have unloaded (“desahogado”) everything…in my work life.” One participant summarized feeling “good because you opened up my memory, which is good for me.” Some participants said that the LHC instrument made it easier to remember past events from their life in the field (“el campo”), particularly their subsistence-farming experiences in Central America/Mexico prior to migrating to the US.

Some participants commented that the act of reflecting on past agrichemical exposures was educational. One participant commented that completing the LHC “helped me to see how exposure to pesticide has consequences”. Another reflected that in Guatemala she never worked with personal protective equipment (PPE), whereas in the US she has more access to PPE. After completing the LHC, another participant commented that “one should take care of oneself around pesticides”.

Participants also linked their experiences of completing the LHC to the greater good. Several commented that migrants suffer wage theft and humiliation and that they are accustomed to keeping quiet and not speaking openly about their experiences. One participant remarked that “Many people are not interested in our work experience or what we’ve gone through”. Several remarked that completing the LHC was their testimony (“testimonio”) and that it was good that this information was going to a university. One participant commented on the direct utility of the LHC, commenting that “you could use this calendar to measure experiences with women who have had different work experiences because everyone’s luck is different in life”.

### 3.5. Qualitative Findings—IPWIDs

Themes that emerged from the LHC administrator assessments included aspects of the LHC that participants used to anchor their recall of specific substances used, comments regarding problems with and suggested improvements for the electronic modality of administration, comments regarding suggested instrument improvements, and comments regarding participant fatigue.

For anchors that assisted with participant recall, administrators noted that the location where the participant lived (appearing at the top of the LHC) assisted participants in delineating their use of specific substances at various points during the lifespan. Providing information about specific interpersonal relationships was also described as an anchoring device. LHC administrators noted that if participants were looking at the LHC form as it was being filled out, this assisted with recall. Some participants looked elsewhere or sat too far away from the instrument. Some interviewers described participants reading the names of each substance that appeared on the LHC along with the administrator as the interview proceeded. Some administrators described participants as having tactile interactions with the paper-based LHC and/or pointing at the LHC to indicate when a particular substance was used.

For suggested LHC improvements, many administrator comments were focused on ways to improve the electronic modality of administration. Suggestions were made to use a bigger monitor/screen so that the participant could clearly see the entire LHC as opposed to the interviewer scrolling through different sections of the LHC on the computer and to be able to enlarge the LHC, given the small appearance of the text on the electronic modality. Administrators struggled with adding lines or amending the electronic LHC when substances that could not be readily classified were mentioned by the participant on the electronic modality. This contrasted with the paper-based modality, where additional substances were added to the LHC and color-coded to indicate which years the participant used the additional substance mentioned. One administrator generated calendar-years based on the participant’s age at the top of the electronic LHC as the participant was using calendar-years (and not age) to delineate years of substance use. Administrators mentioned participants referencing calendar-years with the paper-based LHC, and it was suggested that there be a way to delineate calendar-years along with age prior to collecting data on the LHC form. Some noted that participants wanted to tell additional stories and add context to events beyond the items/questions listed on the LHC form. Some also described participant fatigue occurring as a result of the length of the interview. 

LHC participants described a range of perceptions regarding the LHC. Some felt neutral about the experience, while others described feeling that the LHC was a reflective activity and/or offered lessons that could inform future projects and/or that should inform the way other treatment providers on the reservation interview clients. Some participants described a range of emotions after completing the LHC.

Several participants described neutral feelings following the interview and provided comments such as “I feel ordinary”, “It’s just proof that I was here”, and “Okay”. Others described the LHC as a reflective experience. One participant described how the experience of completing the interview “Makes me aware of the amount of time I’ve been doing things. Puts stuff in perspective for me”. Another participant described how “It was helpful to look at my past use. It makes me not want to be like that again”. Others noted that it helped them understand times when their substance use was relatively better or worse. For example, one participant described how it “Felt good to be able to see it all. How long I have used it and the times I have stopped”.

Some participants mentioned ways in which the LHC could be useful beyond the research activity. Some mentioned that the LHC instrument should be shared with treatment counselors at the local substance-use treatment facility. For example, one participant described how “honestly about time someone came up with this—they need to bring this to (name of local treatment facility)”. Another participant described how “I feel that it’s something little—down the road it could be bigger and more helpful for my people”.

In addition to directly reflecting on the LHC, some participants described their immediate emotional state after completing the LHC. Emotional states described by participants included sadness, gratefulness, a sense of catharsis, feeling good, and feeling that the activity was helpful. Some described never having spoken with anyone before about the information collected by the LHC. For example, one participant described how “I feel lighter, let weight off my shoulders, said some things I haven’t said in a while, it takes the weight off”. Another participant described that “I feel a little bit emotional but grateful and blessed to have done this survey. It’s a reminder of what’s important”.

## 4. Discussion

In this mixed-quantitative-and-qualitative-methods process evaluation of two LHCs administered across two pilot studies with foreign-born agricultural workers and IPWIDs in the US, we have reported findings that can inform the instrumentation and administration of LHCs. The considerations center on LHC-instrument design, administration, ways that within-group heterogeneity may affect the process of LHC administration and understanding the experiences of study participants with LHCs. The results can directly inform future research that plans to implement LHCs as a data-collection method within these populations as well as indicate ethical issues to consider in designing/administering future LHCs.

First, agricultural workers showed greater preference for a paper-based LHC, while preferences for paper versus electronic modes of administration were more evenly distributed among IPWIDs. Qualitative findings included LHC administrators underscoring the utility of visual aspects of the paper-based LHC, including the use of color-coded lines for participants with low literacy levels. LHCs have previously been used in health research with agricultural workers and have been recognized for their utility in aiding memory recollection when working with populations with low literacy levels and/or with very complex and extensive work histories [34,35,36]. While electronic modes of LHC administration may be more efficient and less cumbersome to field, they may make it difficult for participants to visualize the calendar and anchor responses to previous items. In cases where the participant may not be able to visualize the screen, it is important to consider why an LHC instrument is being used as opposed to another modality (e.g., questionnaire) for collecting data with a given population.

Our results suggest that LHC-administrator training and the careful development of a companion interview guide are important for eliciting and documenting information from participants. In both agricultural workers and IPWIDs, over half of each sample reported that the LHC made it easier to remember past events. While these findings are concerned with aspects of LHC administration, they are informative with respect to broader considerations regarding the validity of LHCs [1]. In one study that compared occupational recall via an icon-based LHC versus a traditional questionnaire, the median ratio of jobs reported via questionnaire to the icon-based LHC decreased with distance in time from the interview date, with more jobs being reported during earlier periods of life when measured via LHC [34]. In a separate study of test–retest reliability by LHC, there were higher levels of inter-questionnaire agreement for particular crops and work locations but lower levels of agreement for specific tasks [35]. Previous attempts have also been made to assess LHC accuracy using external validators, such as corroborating the time a chemical was reported as being used with the time the chemical became available on the market [37]. More research is needed, however, to validate measures of past exposure measured via LHC with biomarkers of exposure corresponding to the period in question.

Qualitative findings illustrated that participants raised issues directly concerning the ethical considerations of LHCs. When participants in the current study perceived their participation as contributing to the greater good, this perception may be characterized as a utilitarian ethical reflection [38]. In another study of 37 people who injected drugs, the authors reported mixed emotions among LHC participants, including the utilitarian tradeoff of pleasure at viewing the life in a visualized grid counterbalanced by pain associated with the contents of the grid [21]. It is particularly notable that some of the agricultural workers described their completion of the LHC as having provided their testimonio (“testimony”). In Latin America, testimonios are one of the methods used for making sense of oral histories that produce a narrative of political oppression [39]. They are also understood to disrupt epistemological distances between interviewee and interviewer in research and reissue some power back to the speaker [40,41]. While collecting testimonios was not a method used in this this study, participants’ perceptions of having provided data that may be helpful to others may be useful to consider in future LHCs. Providing open-ended space for reflection and/or testimonies following the completion of LHCs may help participants better process and reflect on their experience after reporting on specific items to an interviewer. 

Finally, some participants described the LHC as providing reflective and quasi-therapeutic moments. The extant literature has explored the qualitative research interview process as cathartic for emotional release, as a method for finding a sense of purpose, empowerment, and healing [42], and as a self-reflexive activity [43]. Responses to the life-history timeline juxtapose emotions of pleasure and pain and can provide an opportunity to create connections and reflect on life events [21], while simultaneously offering some therapeutic value [44]. The comments participants shared in our study align closely with these ideas, with IPWID participants reporting conflicting emotions (“I feel a little bit emotional but grateful and blessed…”) and liberation (“I feel lighter, let weight off my shoulders…”) after completing the survey. The opportunity for self-reflection can allow for transformative effects [45], as described by some agricultural workers in our study who said that the process was educational and could be used among other worker populations to establish connections between occupational hazards and outcomes. Regarding the interview process, maintaining a professional relationship between the LHC administrator and the study participant is vital to ensure that ethical boundaries are not transgressed [45]. Some studies have described the importance of developing the timeline of life events together with a “joint construction of meaning” [46] and a product that is “co-constituted between participant and researcher” [21]. It is within this joint creation of a life-history calendar that the process can become reflective to further enhance therapeutic effects for the participants, while ensuring that the administrator maintains a role as researcher and not as therapist [47]. As summarized by Morecroft et al. (2004), the formation of a participant’s narrative and the ability for the participant to share their experiences with minimal prompting from the researcher is what allows for the most meaningful engagement [48]. Based on the commentaries from both administrators and participants in our study, the findings suggest that participating in the completion of the LHCs was cathartic, may have had some therapeutic value, and provided a meaningful space for reflection.

### Limitations

This study has several limitations. First, the results were based on 81 LHCs conducted in two distinct populations. The sample size limited the number of elements that could be considered in the process evaluation for each population, particularly concerning LHC-administrator characteristics. It is possible that LHC-administrator characteristics (e.g., language dialect, gender, personal familiarity with chemicals remembered in each population, and interpersonal power dynamics) could have impacted the retrospective assessment. The strengths of the LHC highlighted in this study, namely, the visual aspects of the LHC, cannot be generalized to populations or participants with visual impairments. Finally, no biomarkers of exposure were used to cross-validate findings. 

## 5. Conclusions

In this mixed-quantitative-and-qualitative-methods study of LHC administration in foreign-born agricultural workers and IPWIDs in the United States, factors related to instrument design, administration, and ethical considerations were identified. Ample data-collector training and the design of concise interview companion guides were identified as important factors to consider prior to implementing LHCs. The process of administration could be better facilitated by LHC forms that are visible to the participant, particularly in populations with limited literacy. Participants offered ethical reflections regarding how their participation in the research activity contributed to the greater good, and some described participating in the activity as a reflective and cathartic experience. Future iterations of the LHC may benefit from providing free-form and open-ended spaces for participants to reflect on the LHC activity following LHC administration. Further research on the validity of retrospective assessment via LHCs would provide information for the improvement of processes of LHC administration. 

## Figures and Tables

**Table 1:** Duration and ease of LHC administration relative to the demographic characteristics of the participants of the two pilot studies, n = 81.

		Minutes to Complete LHC		Administrator-Assessed Ease of Administration		Participant-Assessed Ease of Administration	
Demographic Characteristics	N	Mean (SD)	F	Mean (SD)	F	%	Chi-Square
IPWIDs (n = 40)							
Gender of participant							
Male	22	45.0 (14.6)	0.4	2.8 (1.5)	0.2	63.6%	0.04
Female	18	48.2 (16.0)		3.1 (2.9)		66.7%	
Tribal recognition status							
Associated tribal member	5	51.8 (20.4)	0.7	2.4 (1.1)	0.4	40.0%	1.6
Registered member of a federally recognized tribe	35	45.7 (14.4)		3.0 (2.3)		68.6%	
Age of participant							
<40 years of age	23	43.2 (12.6)	2.7	43.2 (12.6)	0.5	73.9%	1.9
≥40 years of age	17	50.9 (17.3)		50.9 (17.3)		52.9%	
Foreign-born agricultural workers (n = 41)							
Gender of participant							
Male	14	63.8 (21.2)	0.3	5.4 (2.9)	1.1	57.1%	0.1
Female	27	67.3 (18.9)		4.5 (2.7)		51.9%	
Language of participant							
Monolingual Spanish	25	61.6 (17.7)	3.7	4.6 (2.9)	2.7	52.0%	0.1
Bilingual	16	73.3 (20.7)		5.1 (2.6)		56.3%	
Age of participant							
<60 years of age	35	65.9 (19.1)	0.0	4.7 (2.8)	5.2	51.4%	0.5
≥60 years of age	6	67.5 (23.6)		5.7 (2.8)		66.7%	
Country of origin of participant							
Mexico	22	65.5 (19.4)	1.0	3.9 (2.4)	3.1	63.6%	2.7
Guatemala	18	68.3 (19.7)		5.7 (2.9)		44.4%	
Honduras	1	40.0 (0.0)		8.0 (0.0)		0.0%	

**Table 2:** Human–chemical interactions documented over the lifespan relative to the demographic characteristics of the participants of the two pilot studies, n = 81.

		Total Number of Drugs Used during Lifetime		Cumulative Person-Polysubstance-Years of Use Documented	
Demographic Characteristics	N	Mean (SD)	F	Mean (SD)	F
IPWIDs (n = 40)					
Gender of participant					
Male	22	6.7 (2.1)	0.17	68.5 (25.5)	0.45
Female	18	6.4 (2.3)		75.1 (36.6)	
Tribal recognition status					
Associated tribal member	5	6.6 (2.2)	0.14	57.6 (31.0)	1.2
Registered member of a federally recognized tribe	35	6.2 (2.3)		73.4 (30.6)	
Age of participant			0.06		15.3
<40 years of age	23	6.5 (2.1)		57.4 (27.6)	
≥40 years of age	17	6.6 (2.3)		90.4 (24.4)	
		**Cumulative Person-Years of Labor Documented**		**Cumulative Person-Years of Exposure to Agrichemicals Documented**	
	**N**	**Mean (SD)**	**F**	**Mean (SD)**	**F**
Foreign-born agricultural workers (n = 41)					
Gender of participant					
Male	14	40.1 (17.3)	3.0	59.1 (49.3)	1.0
Female	27	32.3 (11.5)		46.6 (31.5)	
Language of participant					
Monolingual Spanish	25	39.2 (14.9)	6.7	51.6 (39.7)	0.0
Bilingual	16	28.3 (9.8)		49.8 (37.3)	
Age of participant					
<60 years of age	35	31.3 (11.6)	27.3	45.9 (32.5)	4.5
≥60 years of age	6	56.5 (4.4)		80.2 (58.2)	
Country of origin of participant					
Mexico	22	38.5 (13.6)	3.6	40.1 (25.8)	10.7
Guatemala	18	29.4 (12.7)		56.7 (37.3)	
Honduras	1	56.0 (0.0)		185.0 (0.0)	

**Table 3:** Substances reported by modality of LHC administration (paper versus electronic) in a sample of Indigenous people who used injection drugs, n = 40.

	Paper-Based LHC (n = 20)			Electronic LHC (n = 20)				
Substance	No. Detected	Years of Reported Use ^a^		No. Detected	Years of Reported Use ^a^		Chi-Square Test	Wilcoxon Rank-Sum |Z|
	N (%)	Mean	SD	N (%)	Mean	(SD)		
**CNS Depressants**								
Alcohol	20 (100%)	20.7	10.0	20 (100%)	21.7	9.2	0.00	0.64
Sedatives/hypnotics/tranquilizers	4 (20%)	1.0	3.0	5 (25%)	0.8	2.4	0.14	0.26
Barbiturates	0 (0%)	0.0	0.0	1 (5%)	0.2	0.7	1.03	1.00
**CNS Stimulants**								
Cocaine	10 (50%)	1.4	2.4	11 (55%)	4.6	8.4	0.10	0.88
Amphetamines	7 (35%)	2.9	6.8	8 (40%)	3.3	7.4	0.11	0.36
Methamphetamines	19 (95%)	14.3	8.7	19 (95%)	14.0	7.8	0.00	0.11
**Hallucinogens**	10 (50%)	1.0	1.4	10 (50%)	1.3	2.2	0.00	0.24
**Narcotic Analgesics**								
Heroin	6 (30%)	0.7	1.3	7 (35%)	0.6	1.0	0.11	0.16
Suboxone	11 (55%)	1.0	1.6	6 (30%)	0.3	0.5	2.56	1.82
Methadone	3 (15%)	0.5	1.6	5 (25%)	0.8	1.5	0.63	0.83
Other opiates/analgesics	15 (75%)	5.2	6.0	13 (65%)	2.8	5.4	0.48	1.28
**Inhalants**	9 (45%)	1.5	3.1	5 (25%)	0.6	1.2	1.76	1.34
**Cannabis**	18 (90%)	19.3	11.2	20 (100%)	23.2	12.1	2.11	0.92

Abbreviations: ASI, Addiction Severity Index; LHC, life-history calendar; CNS, central nervous system; SD, standard deviation; P, probability value. ^a^ Among participants reporting any use.

## Data Availability

The data presented in this study are available on request from the corresponding author. The data are not publicly available due to privacy concerns.
